# Nanostructured Lipid Carriers Enhance Ciprofloxacin Antibacterial Activity Through Diffusion-Controlled Release and Modulation of Bacterial Growth Kinetics

**DOI:** 10.3390/pharmaceutics18040496

**Published:** 2026-04-17

**Authors:** Javiera Carrasco-Rojas, Felipe I. Sandoval, Javiera Solas-Soto, Christina M. A. P. Schuh, Lorena Rubio-Quiroz, Carlos F. Lagos, Francisco Arriagada, Andrea C. Ortiz

**Affiliations:** 1 Departamento de Ciencias y Tecnología Farmacéutica, Facultad de Ciencias Químicas y Farmacéuticas, Universidad de Chile, Santiago 8380494, Chile; javiera.carrasco@uchile.cl; 2 Centro de Medicina Regenerativa, Facultad de Medicina, Clínica Alemana-Universidad del Desarrollo, Santiago 7550000, Chile; felipe.sandoval@udd.cl (F.I.S.); cschuh@udd.cl (C.M.A.P.S.); 3 Escuela de Química y Farmacia, Facultad de Ciencias, Universidad San Sebastián, Santiago 7510157, Chile; jsolass@correo.uss.cl (J.S.-S.); lrubioq@docente.uss.cl (L.R.-Q.); carlos.lagos@uss.cl (C.F.L.); 4 Centro Basal Ciencia & Vida, Fundación Ciencia & Vida, Av. del Valle Norte 725, Santiago 8580702, Chile

**Keywords:** nanostructured lipid carriers, ciprofloxacin, antibacterial activity, bacterial growth kinetics, controlled drug release

## Abstract

**Background:** The increasing prevalence of multidrug-resistant bacterial infections highlights the need for drug-delivery strategies that improve antimicrobial exposure and sustain therapeutic activity. In this study, ciprofloxacin-loaded nanostructured lipid carriers (NLC-CIP) were developed and evaluated to better understand how formulation-dependent release behavior influences antibacterial performance against *Escherichia coli*. **Methods:** NLC-CIP were prepared and characterized in terms of size, polydispersity, encapsulation efficiency, and colloidal stability. In vitro release profiles were evaluated across different pH conditions, followed by kinetic modeling. Stability under refrigerated storage was assessed. Antibacterial performance was determined through IC_50_ measurements and dynamic growth-kinetic analyses, while cytotoxicity was evaluated in HepG2 cells. **Results:** Ciprofloxacin incorporation increased hydrodynamic diameter (~116 to 194 nm) while preserving low polydispersity (PdI~0.04), high colloidal stability, and encapsulation efficiency (96%). Release studies showed medium-dependent behavior, with rapid release at pH 1.2, 4.5, and 7.4, and more sustained profile at pH 6.8, consistent with diffusion-controlled kinetics (Weibull model). Refrigerated storage preserved release profiles while slowing early-stage kinetics. NLC-CIP showed improved apparent antibacterial activity, reducing the IC_50_ from 4.9 to 1.2 ng/mL, and sustained bacterial suppression by decreasing growth rates and prolonging doubling times. Unloaded NLCs showed no antibacterial activity, and cytotoxicity assays confirmed favorable biocompatibility. **Conclusions:** Overall, these results show that NLC-based encapsulation can modulate ciprofloxacin release and reshape drug exposure over time, thereby improving antibacterial performance under the tested conditions. This study supports integrated release and growth-kinetic analyses as a more informative framework for evaluating lipid-based antibiotic delivery systems.

## 1. Introduction

The global rise of multidrug-resistant (MDR) bacteria represents a major public health challenge and threatens the long-term effectiveness of current antibiotic therapies [1]. From a pharmaceutical sciences perspective, therapeutic failure in bacterial infections is not solely driven by microbial resistance mechanisms, but also by limitations in dosage form performance, including inadequate drug exposure at the site of infection and limited ability of conventional dosage forms to sustain therapeutically relevant concentrations over time [2]. Suboptimal pharmacokinetic profiles and poorly sustained local drug levels often necessitate prolonged treatment regimens, increasing the risk of systemic toxicity and contributing to overall healthcare burden [3]. These considerations highlight the need for advanced drug-delivery strategies capable of modulating drug release and maintaining effective exposure over clinically relevant time scales.

*Escherichia coli* (*E. coli*) is one of the most clinically relevant Gram-negative pathogens and a leading cause of community- and healthcare-associated infections worldwide [4]. Pathogenic strains of *E. coli* are responsible for a wide range of diseases, including urinary tract infections, bloodstream infections, neonatal meningitis, and severe gastrointestinal disorders, with certain variants associated with life-threatening complications such as hemolytic uremic syndrome [5]. The increasing prevalence of MDR and extended-spectrum β-lactamase (ESBL)-producing *E. coli* strains has further complicated treatment and narrowed available therapeutic options, contributing to higher morbidity and mortality [6]. In this context, delivery systems capable of maintaining effective and sustained antimicrobial concentrations at infection sites, while mitigating exposure-related limitations that contribute to reduced antibacterial effectiveness, are of particular pharmaceutical interest.

Ciprofloxacin, a widely prescribed fluoroquinolone, remains a key therapeutic agent for the treatment of infections caused by Gram-negative bacteria, including *E. coli* [7]. Its broad-spectrum activity, favorable tissue penetration, and well-established clinical efficacy have positioned it as a cornerstone antibiotic in clinical practice [8]. However, increasing resistance, efflux-mediated reductions in intracellular drug accumulation, and limited ability to sustain effective local concentrations at specific infection sites, can significantly compromise therapeutic outcomes [9]. From a formulation standpoint, strategies that enhance local ciprofloxacin availability and sustain exposure through controlled-release systems represent an attractive approach to improving antibacterial performance without altering the molecular structure of the drug.

Nanostructured lipid carriers (NLCs) have emerged as versatile, scalable, and biocompatible platforms for drug delivery, including antimicrobial applications [10]. As pharmaceutical dosage forms, NLCs are characterized by partially crystalline lipid matrices that enable high drug-loading capacity, improved physical stability, and controlled release kinetics, while protecting incorporated drugs from premature degradation [11]. These attributes can be exploited to modulate antibiotic exposure profiles and, in turn, antibacterial performance [12,13]. However, despite numerous reports describing ciprofloxacin-loaded or antibiotic-loaded lipid nanoparticles, most studies follow a conventional framework centered on physicochemical characterization, endpoint antibacterial measurements, such as minimum inhibitory concentration (MIC) values or static viability assays [14]. While informative, these approaches provide limited insight into how formulation-dependent release behavior relates to the temporal dynamics of bacterial growth and inhibition. From a pharmaceutical performance and engineering perspective, the relationship between drug-release kinetics from nanoscale carriers and real-time bacterial growth dynamics remains insufficiently defined. This gap limits mechanistic interpretation and makes it difficult to distinguish whether improved antibacterial outcomes arise mainly from sustained release, altered drug exposure over time, or other formulation-related effects. Addressing this limitation requires integrated evaluation strategies that move beyond static potency metrics and directly relate release behavior to dynamic biological response. In this context, the present study was designed to provide a more detailed pharmaceutical understanding of how a ciprofloxacin-loaded NLC system influences drug performance, particularly by relating release behavior to the temporal dynamics of bacterial growth.

In this study, we developed ciprofloxacin-loaded nanostructured lipid carriers (NLC-CIP) and conducted a comprehensive physicochemical and biological evaluation focused on formulation performance. The nanoparticles were characterized in terms of size distribution, morphology, colloidal stability, and encapsulation performance, alongside a detailed kinetic analysis of ciprofloxacin release under different pH conditions. Antibacterial activity against *E. coli* (ATCC 25922) was assessed using both dose–response experiments and real-time growth-curve analyses to capture dynamic effects on bacterial proliferation. Additionally, cytotoxicity was evaluated in HepG2 cells to assess formulation biosafety. By integrating release kinetics with bacterial growth dynamics, this study provides a more mechanistic and formulation-oriented assessment of antibacterial performance, supporting the design of NLC-based dosage forms for antibiotic delivery.

## 2. Materials and Methods

### 2.1. Materials

Gelucire^®^ 44/14 was kindly provided by Gattefossé (Saint-Priest, France). Miglyol^®^ 812, Tween^®^ 80, ciprofloxacin (≥95% purity, HPLC), dimethyl sulfoxide (DMSO, molecular biology grade), 3-(4,5-dimethylthiazol-2-yl)-2,5-diphenyltetrazolium bromide (MTT, ≥97.5% purity), phosphotungstic acid hydrate, trehalose dihydrate, tryptic soy broth (TSB), Amicon^®^ Ultra centrifugal filters (10 kDa MWCO), agar-agar, and triple sugar iron (TSI) medium were obtained from Merck (Merck KGaA, Darmstadt, Germany). SnakeSkin^TM^ Dialysis Tubing (cellulose membrane, 10 kDa MWCO) was purchased from Thermo Fisher Scientific (Carlsbad, CA, USA). Yeast extract and tryptone were purchased from Winkler (Milan, Italy). Acetic acid (HPLC grade), acetonitrile (HPLC grade), and sodium chloride were acquired from PanReac AppliChem ITW Reagents (Darmstadt, Germany). Dulbecco’s Modified Eagle Medium (DMEM, high-glucose), phosphate-buffered saline (PBS, without calcium and magnesium), fetal bovine serum (FBS), and penicillin/streptomycin solution were obtained from Cytiva (Marlborough, MA, USA). Trypsin-EDTA solution (0.25%) was purchased from Gibco (Waltham, MA, USA). Ultra-pure water (resistivity 18.2 MΩ·cm) was generated using a Simplicity System (Millipore, Burlington, MA, USA). All reagents were used as received without further purification.

### 2.2. Preparation of Nanostructured Lipid Carrier (NLC)

**Figure 1:**
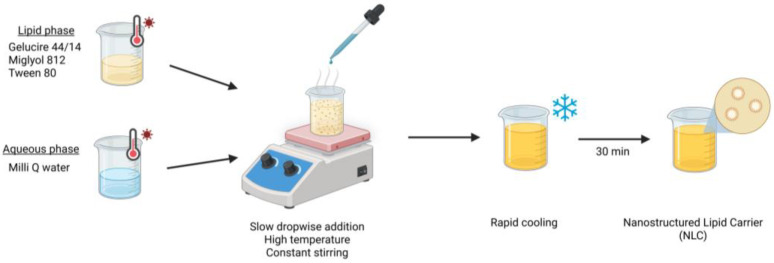
Schematic representation of the NLC fabrication process.

The fabrication process of the nanostructured lipid carrier (NLC) has been described in a previous study and is schematically illustrated in Figure 1 [15]. The nanosystem was prepared using a hot emulsification method with slow dropwise addition followed by cooling. The NLC formulation comprises Gelucire^®^ 44/14 (3.3% *w*/*v*), Miglyol^®^ 812 (1.2% *w*/*v*), and Tween^®^ 80 (2.5% *w*/*v*) as the lipid phase, with water constituting the aqueous phase (final volume: 30 mL). Briefly, the lipid phase was blended and heated to 85 °C. In parallel, the aqueous phase was heated to 85 °C and added dropwise to the lipid phase under continuous stirring at 400 rpm. The resulting mixture was rapidly cooled to 4 °C and held for at least 30 min without stirring. For ciprofloxacin-loaded NLC (NLC-CIP), 50 mg of ciprofloxacin (CIP) was incorporated into the lipid phase prior to emulsification, following the same preparation procedure.

### 2.3. Hydrodynamic Diameter, Polydispersity Index, Zeta Potential and Nanoparticle Concentration Evaluation

Hydrodynamic diameter (HD), polydispersity index (PdI), and zeta potential (ZP) were measured using a Zetasizer Nano ZS (Malvern Panalytical Ltd., Malvern, UK). HD and PdI were determined by dynamic light scattering (DLS), while ZP was assessed by laser Doppler microelectrophoresis. Samples were diluted 1:10 (*v*/*v*) in water and analyzed in triplicate, with each measurement representing the mean of eleven runs. Instrument parameters were set as follows: medium viscosity 0.08872 cP, sample refractive index 1.333, wavelength 633 nm, detection angle 173°, and equilibration time 120 s. Disposable cuvettes and folded capillary cells were used for HD and ZP determinations, respectively. On the other hand, nanoparticle concentration was evaluated by nanoparticle tracking analysis (NTA) using a NanoSight NS300 (Malvern Panalytical Ltd., Malvern, UK) equipped with a 532 nm diode laser (green). Diluted samples were introduced into the measurement chamber with sterile syringes. Data were acquired at room temperature over 90 s in single shutter and gain mode, with manual adjustments of shutter, gain, brightness, and threshold.

### 2.4. Morphology of NLC

The morphology of NLC and NLC-CIP was examined by transmission electron microscopy (TEM) using an Inspect F50 Scanning Transmission Electron Microscope (FEI, Hillsboro, OR, USA). Samples were diluted 1:100 in water, deposited onto film-coated copper grids, stained with 1.0% (*w*/*v*) phosphotungstic acid for 2 min, rinsed with water, and air-dried at room temperature for 48 h prior to imaging.

### 2.5. Encapsulation Efficiency and Drug Loading of Ciprofloxacin

To determine the quantities of drugs encapsulated within the nanostructured lipid carriers (NLCs), the formulations were subjected to ultracentrifugation using Amicon^®^ Ultra filters with a 10,000 Da molecular weight cut-off (MWCO) at 6000× *g* for 15 min. “Free ciprofloxacin” is defined as the non-encapsulated drug fraction, operationally determined as the fraction that passes through the Amicon^®^ Ultra 10 kDa MWCO membrane under the applied centrifugation conditions. Subsequently, the filtrate (free ciprofloxacin) was collected and quantified by high-performance liquid chromatography (HPLC).

CIP quantification was performed using the analytical method described by Wu et al. [16]. Chromatographic analyses were conducted on an Alliance 2695 HPLC system (Waters Corporation, Milford, MA, USA). Separation was achieved using an Inertsil ODS-3 C18 column (4.6 mm × 250 mm, 5 μm; GL Science Inc., Tokyo, Japan). The mobile phase consisted of acetonitrile and 2% (*v*/*v*) acetic acid in a ratio of 16:84 (*v*/*v*), delivered at a flow rate of 1.0 mL/min. The injection volume was 10 μL, and ciprofloxacin was monitored at 280 nm.

The encapsulation efficiency (% EE, Equation (1)) and drug-loading capacity (% DL, Equation (2)) of ciprofloxacin were calculated according to the following equations:

(1) $$\%EE=\frac{\left({drug}_{total}-{drug}_{free}\right)}{{drug}_{total}}*100$$

(2) $$\%DL=\frac{{drug}_{total}-{drug}_{free}}{solidlipid+({drug}_{total}-{drug}_{free})}*100$$

where drug_total_ and drug_free_ represent the initial amount of CIP added to the formulation and the amount that remained unencapsulated in the NLC-CIP system, respectively.

### 2.6. Calorimetric Evaluation of NLC

The physical state of NLC and NLC-CIP components was analyzed by differential scanning calorimetry (DSC) using a DSC131 instrument (SETARAM Inc., Cranbury, NJ, USA). Prior to analysis, NLC dispersions were freeze-dried with 5% trehalose (1:1) for 48 h. Each sample (1–5 mg) was sealed in aluminum pans and heated from 25 °C to 300 °C at 5 °C/min under nitrogen atmosphere.

### 2.7. In Vitro Drug Release Studies

CIP release from the NLC formulation was evaluated using the dialysis bag method [17]. To maintain sink conditions and ensure adequate drug solubility across the experimental range, the release media (pH 1.2, 4.5, 6.8, and 7.4) were supplemented with 1% (*w*/*v*) Tween^®^ 80. These pH values were selected to represent the typical pH conditions encountered along the gastrointestinal tract transit (1.2, 4.5, and 6.8) and typical physiological/systemic conditions (7.4). SnakeSkin™ dialysis tubing (10 kDa MWCO) was immersed in 10 mL of release medium maintained at 37 °C with continuous stirring at 100 rpm. At predetermined time points over 72 h, 1.5 mL aliquots were withdrawn and replaced with fresh medium. CIP concentrations were quantified in triplicate by HPLC, as previously described in Section 2.5.

The in vitro release profiles of CIP from the NLC formulation were analyzed using several kinetic models to elucidate the underlying release mechanisms and rate-controlling steps. For full profile, the cumulative fraction of ciprofloxacin released (F_t_/F_∞_) was normalized to the total drug amount released at 72 h, which was considered the asymptotic maximum (F_∞_) as estimated from the Weibull model. Additionally, to enable a more physiologically relevant kinetic interpretation, release data were further analyzed within time windows corresponding to gastrointestinal transit times. Specifically, a 2 h window was considered for pH 1.2 (gastric phase), followed by a 2 h transition phase at pH 4.5, and a 6 h window at pH 6.8 representing residence in the small intestine. Within each pH-specific window, release profiles were normalized to the cumulative amount of drug released during that interval, allowing isolation of the dominant release kinetics under each simulated physiological condition and minimizing bias from early burst effects. Release data were fitted to five commonly applied mathematical models for lipid-based nanocarriers: Weibull (Equation (3)), power law (Korsmeyer–Peppas) (Equation (4)), Higuchi (Equation (5)), zero-order (Equation (6)), and first-order (Equation (7)) models [18].

(3) $$\frac{F_{t}}{F_{\infty }}=100\cdot \left(1-e^{-\frac{t^{\beta }}{\alpha }}\right)$$

(4) $$\frac{F_{t}}{F_{\infty }}=k_{KP}\cdot t^{n}$$

(5) $$\frac{F_{t}}{F_{\infty }}=k_{H}\cdot t^{0.5}$$

(6) $$\frac{F_{t}}{F_{\infty }}=k_{0}\cdot t$$

(7) $$\frac{F_{t}}{F_{\infty }}=100\cdot \left(1-e^{-k_{1}\cdot t}\right)$$

where k_KP_, k_H_, k_0_, and k_1_ are the Korsmeyer–Peppas, Higuchi, zero-order, and the first-order release apparent rate constants, respectively. In the Korsmeyer–Peppas model, the diffusional exponent *n* characterizes the release mechanism: if *n* = 0.43 indicates a Fickian diffusion, when 0.43 < *n* < 0.85 it is anomalous transport (non-Fickian diffusion), and if *n* ∼ 0.85, it indicates case-II transport [19]. For the Weibull model, α is the scale parameter and β is the curve shape factor, which is often correlated with the dominant release mechanism described by the *n* value in the Korsmeyer–Peppas model [20,21]. Particularly, when β > 1.0 indicates a sigmoidal profile (complex or multi-step release), β = 1.0 corresponds to an exponential profile (first-order release), and β < 1.0 yields a parabolic curve (Fickian diffusion when β ≤ 0.75, or anomalous transport when 0.75 < β < 1.0).

### 2.8. Post-Storage Evaluation

After storage at 4 °C for 8 weeks, the formulation was evaluated in terms of hydrodynamic diameter, polydispersity index (PdI), zeta potential, encapsulation efficiency, and in vitro drug release, using the same methodologies previously described for the freshly prepared formulation.

### 2.9. Bacterial Strain, Growth Conditions, and Antibacterial Activity Evaluation

For microbiological experiments, the reference strain *E. coli* ATCC 25922 (American Type Culture Collection) was employed. Bacterial stocks were preserved at −80 °C and routinely propagated on Luria–Bertani (LB) agar medium prepared with sodium chloride, agar, yeast extract, and tryptone, at final concentrations of 0.01, 0.015, 0.005, and 0.01 g/mL, respectively. The medium was adjusted to pH 7.0 and cultures were incubated at 37 °C under aerobic conditions.

To assess the antibacterial potential of NLC, NLC-CIP, and CIP, overnight cultures were adjusted to a turbidity equivalent of 0.5 McFarland standard using a DensiCheck Plus turbidimeter (BioMérieux, Marcy-l’Étoile, France) and transferred into sterile 5 mL Falcon round-bottom polystyrene tubes (Corning Inc., Corning, NY, USA). The resulting suspension corresponded to approximately 1.5 × 10^7^ colony-forming units (CFUs), which were distributed into wells of sterile 96-well plates (NEST Biotechnology, Wuxi, China). Test formulations were added to achieve final concentrations of CIP between 0.025 ng/mL and 500 ng/mL in a final volume of 200 µL of TSB per well. All antibacterial experiments were conducted based on CIP concentration. For CIP, concentrations correspond to the actual drug content. In the case of NLC-CIP, concentrations are expressed as the equivalent CIP content within the formulation. For unloaded NLC, the nanoparticle concentration was adjusted to match the amount of nanoparticles present in each corresponding NLC-CIP condition. Accordingly, results are presented as a function of ciprofloxacin concentration.

Plates were incubated for 18 h at 37 °C under standard oxygen conditions. Bacterial growth was determined spectrophotometrically at 600 nm using a microplate reader (Multiskan Sky, Thermo Scientific, Waltham, MA, USA). Growth inhibition was expressed as a percentage relative to untreated control wells, and dose–response relationships were modeled to calculate the half-maximal inhibitory concentration (IC_50_). All assays were conducted in at least three independent experiments, each including five biological replicates per treatment.

### 2.10. Bacterial Growth Kinetics and Area Under the Curve Calculation

Growth kinetics of *Escherichia coli* ATCC 25922 were assessed in sterile 96-well plates (NEST Biotechnology, China) using a starting inoculum adjusted to 0.5 McFarland standard (≈1.5 × 10^7^ CFU) in TSB medium. Test formulations (NLC, NLC-CIP, and CIP) were applied at final concentrations ranging from 0.25 ng/mL to 500 ng/mL in 200 µL per well. Plates were incubated at 37 °C under aerobic conditions, and bacterial growth was monitored by measuring optical density at 600 nm (OD_600_) every 30 min up to 18 h (overnight) with a microplate reader (Multiskan Sky, USA).

The percentage of NP inhibition was evaluated using OD_600_ data and the following equation:

(8) (%)=ODfbacteria−(ODfNLC+bacteria−ODcontrolNLC−bacteria)ODfbacteria∗100

where ${OD}_{f}^{bacteria}$ is the final optical density of the positive control with bacteria alone; ${OD}_{f}^{NLC+bacteria}$ is the final optical density of bacteria in contact with NLC; and ${OD}_{control}^{NLC-bacteria}$ is the optical density of NLC without bacteria [22].

To quantify the overall bacterial growth under each condition, the area under the growth curve (AUC) was calculated using the trapezoidal rule. Raw OD data were exported to Excel and processed in R software (4.0.0), where AUC values were computed for each treatment group.

### 2.11. Cytotoxicity Assay in HepG2 Cells

The safety profile of NLC, NLC-CIP, and CIP was assessed using the human hepatocellular carcinoma cell line HepG2 (ATCC HB-806), kindly provided by Dr. Carlos Lagos. Cells were routinely maintained in high-glucose DMEM supplemented with 10% fetal bovine serum (FBS) and penicillin–streptomycin, under standard culture conditions (37 °C, 5% CO_2_, humidified incubator).

When cultures reached ~90% confluence, monolayers were rinsed twice with phosphate-buffered saline (PBS) and detached using 0.25% trypsin–EDTA solution for 5 min. Cells were collected by centrifugation at 300× *g* for 5 min and subsequently seeded into 96-well plates (NEST Biotechnology, China) at a density of 1 × 10^4^ cells/well. Treatments with NLC, NLC-CIP, or free ciprofloxacin were applied at concentrations ranging from 0.025 ng/mL to 500 ng/mL.

After 24 h and 48 h of exposure, cell viability was determined using the MTT assay. A final concentration of 0.5 mg/mL MTT was added to each well, followed by 90 min incubation at 37 °C. The resulting formazan crystals were dissolved in dimethyl sulfoxide (DMSO), and absorbance was recorded at 570 nm using a SYNERGY H1 microplate reader (BioTek, Winooski, VT, USA) operated with Gen5 software.

### 2.12. Statistical Analysis

All experiments were performed with at least three independent replicates or three biological experiments, each performed in technical triplicates. Drug release data were fitted using the DDSolver^®^ add-In (Microsoft Excel) program, and the coefficient of determination (R^2^) was used where applicable. Statistical analyses were carried out using two-way ANOVA followed by multiple comparisons. IC_50_ values were calculated by nonlinear regression using a four-parameter logistic model. IC_50_ values were compared using a Mann–Whitney test in GraphPad Prism (version 10.5.0), with *p* < 0.05 considered statistically significant.

## 3. Results and Discussion

### 3.1. Physicochemical Characterization of Nanostructure Lipid Carriers

Regarding the composition of the NLC, Gelucire^®^ 44/14 was selected as the solid lipid due to its multifunctional composition, which enables the formation of a suitable lipid matrix for NLCs while simplifying the manufacturing process by using a single solid lipid component and enhancing drug solubilization.

The physicochemical evaluation of the NLC and NLC-CIP formulations revealed clear differences in hydrodynamic diameter, zeta potential, and particle concentration (Table 1). Unloaded NLCs exhibited an average hydrodynamic diameter of 116.0 ± 2.2 nm, whereas NLC-CIP showed a significantly larger size of 193.9 ± 1.7 nm. This increase is consistent with the incorporation of ciprofloxacin into the lipid matrix, a process that is known to induce lipid rearrangement and expansion of the nanostructure [23]. Despite this size increase, both systems maintained very low polydispersity index values (PdI~0.04), confirming a narrow and uniform size distribution, which is a desirable feature for formulation reproducibility and predictable performance.

CIP loading induced a decrease in the magnitude of the zeta potential, from −4.9 ± 0.2 mV (NLC) to −1.2 ± 0.4 mV (NLC-CIP), reflecting changes in the interfacial electrostatic environment due to drug incorporation. As CIP is predominantly protonated at the formulation pH, partial charge compensation of negatively charged lipid components is expected [24]. However, both systems exhibit low zeta potential values, indicating minimal electrostatic stabilization, with colloidal stability primarily governed by steric effects from PEG-containing lipids in Gelucire^®^ 44/14.

Drug loading led to a decrease in particle concentration, from 1.5 × 10^17^ particles/mL and 9.9 × 10^16^ particles/mL for unloaded NLC and NLC-CIP, respectively. This decrease is most plausibly attributed to the influence of ciprofloxacin on the nucleation-growth dynamics processes during nanoparticle formation. The presence of the ciprofloxacin may partially occupy or perturb the droplet interface, modify effective interfacial tension, or alter the fluidity of the molten lipid phase, thereby reducing the efficiency of droplet breakup during emulsification and promoting early coalescence. These effects result in the formation of fewer and larger particles, consistent with other authors [25,26].

**Figure 2:**
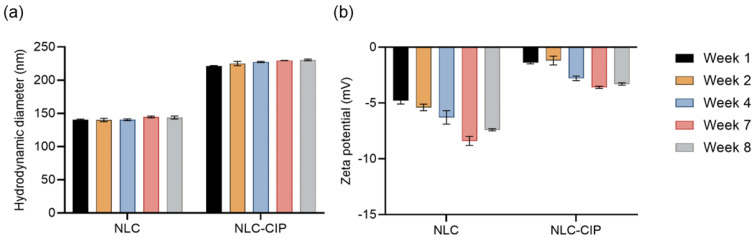
Colloidal stability of (**a**) NLC and (**b**) NLC-CIP at 4 °C, evaluated by monitoring hydrodynamic diameter and zeta potential over time. Data represent mean ± standard deviation from three independent experiments, each performed in triplicate.

Colloidal stability was assessed over eight weeks at 4 °C by monitoring hydrodynamic diameter and zeta potential (Figure 2). Both NLC and NLC-CIP maintained stable and homogeneous size distributions (~140 nm and ~220 nm, respectively) with no significant changes over time. NLCs exhibited slightly increasing negative zeta potential values, reaching ~–8 mV, likely due to lipid rearrangement and exposure of negatively charged fatty acid moieties [27]. In contrast, NLC-CIP showed consistently less negative values (~–2 to –4 mV), with a modest increase in negativity over time, possibly reflecting interfacial drug release and lipid reorganization. Despite near-neutral zeta potential values, no aggregation was observed, indicating that colloidal stability is primarily governed by steric stabilization provided by PEG chains in Gelucire^®^ 44/14.

After 8 weeks of storage at 4 °C, the % EE of ciprofloxacin was high for both freshly prepared and post-storage NLCs, reaching 96% and 94%, respectively. In terms of drug loading (% DL), these formulations contained approximately 5.2% and 4.9%, respectively.

The physicochemical characteristics observed in this study are consistent with previous reports on ciprofloxacin-loaded lipid-based nanosystems. For example, Youssef et al. formulated NLCs containing 0.1% (*w*/*v*) of CIP in a final volume of 10 mL, and reported hydrodynamic diameters ranging from 142 to 380 nm, depending on the Tween^®^ 80 concentration employed [28]. Similarly, Dlamini et al. reported an encapsulation efficiency of approximately 80% (corresponding to around 8 mg of CIP) and hydrodynamic diameter and zeta potential values comparable to those reported here [29]. Overall, CIP incorporation appears to depend on multiple formulation parameters, including component type and ratios. Previous studies report that drug amount and lipid ratio enhance encapsulation efficiency, whereas higher surfactant concentrations reduce loading capacity, likely due to mixed micelle formation or pore generation within the carrier [30,31,32].

**Figure 3:**
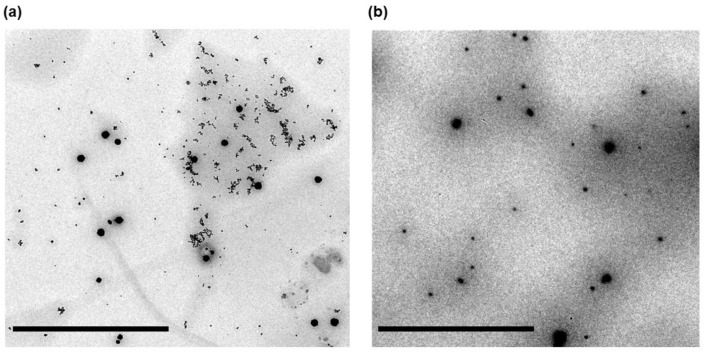
Transmission electron microscopy (TEM) images of (**a**) NLC and (**b**) NLC-CIP. Both formulations exhibit predominantly spherical particles with a relatively uniform size range. Scale bar: 2 µm.

Transmission electron microscopy analysis further confirmed the formation of spherical, well-defined nanoparticles (Figure 3). The particles sizes estimated from TEM images (~90 nm for NLC and ~120 nm for NLC-CIP) were smaller than those measured by DLS, as expected due to the absence of the hydration layer under TEM conditions. Importantly, these values are consistent with the relative size trends observed by light scattering.

### 3.2. Thermal Study of Nanostructured Lipid Carrier

**Figure 4:**
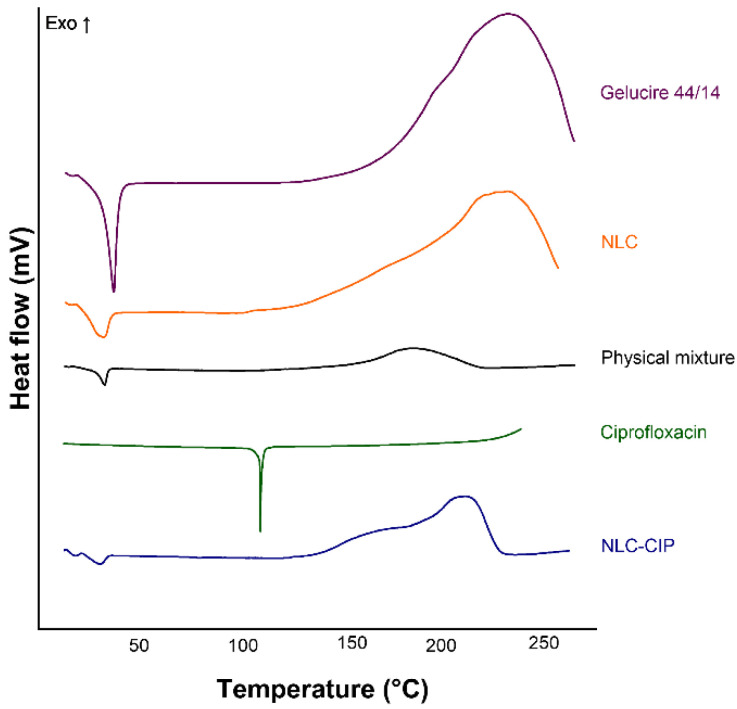
Differential scanning calorimetry (DSC) thermograms of Gelucire^®^ 44/14 (purple line), ciprofloxacin (green line), the physical mixture used for NLC preparation (gray line), and the lyophilized NLC (orange line) and NLC-CIP (blue line) formulations. The curves shown are representative thermograms obtained from repeated measurements.

DSC was employed to gain insight into the physical state of the lipid matrix and the solid-state disposition of ciprofloxacin within the NLC. The thermograms revealed a sharp endothermic peak for Gelucire^®^ 44/14 at approximately 43–46 °C, corresponding to its melting point (Figure 4) [33,34,35]. The NLC formulation exhibited a broader, slightly shifted melting peak with reduced intensity, indicating decreased crystallinity as a result of the presence of liquid lipids and the formation of a partially disordered matrix. This behavior is characteristic of NLCs and is commonly attributed to imperfections within the lipid lattice. Pure CIP exhibited a distinct endothermic transition at approximately 110 °C, consistent with a solid-state thermal event reported for the crystalline drug [36,37]. Notably, this peak was absent in both the physical mixture and the NLC-CIP formulation, where only thermal events corresponding to Gelucire^®^ 44/14 are detectable. The disappearance of the ciprofloxacin peak suggests that the drug is no longer present as a crystalline phase but is instead molecularly dispersed and/or present in an amorphous state within the lipid matrix. Importantly, drug incorporation did not markedly alter the characteristic thermal behavior of the carrier system, indicating that the structural integrity of the lipid matrix is preserved upon loading. The presence of ciprofloxacin in a non-crystalline state is consistent with previous reports on lipid-based nanocarriers and is often associated with enhanced apparent solubility and favorable release behavior [38,39].

### 3.3. In Vitro Release Behavior of Ciprofloxacin from NLC Formulations

The release behavior of CIP from NLC formulations was evaluated across a physiologically relevant pH range (1.2, 4.5, 6.8, and 7.4) for freshly prepared systems (NLC-CIP(F)) and for formulations stored at 4 °C for 8 weeks (NLC-CIP(S)). This design was intended to capture how the formulation responds to changes in the chemical environment, rather than to define a specific route of administration. In particular, this range allows assessment of formulation performance under conditions spanning acidic to near-neutral environments, which is relevant considering the amphoteric nature of ciprofloxacin (pKa ≈ 6.0 and 8.8) and its pH-dependent ionization and solubility. Furthermore, the above conditions allow for the evaluation of the impact of refrigerated storage on drug availability and release kinetics.

**Figure 5:**
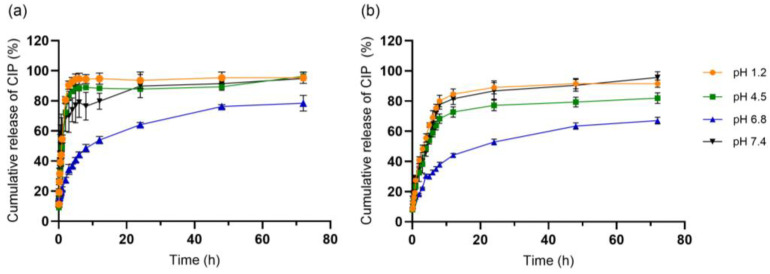
Cumulative ciprofloxacin release (%) from (**a**) freshly prepared NLC-CIP (NLC-CIP(F)) and (**b**) post-storage NLC-CIP (NLC-CIP(S)) formulations at pH 1.2, 4.5, and 6.8 over 72 h at 37.5 °C. Data are presented as mean ± standard deviation from three independent experiments, each performed in triplicate.

Across all tested media, NLC-CIP(F) exhibited a characteristic biphasic profile (Figure 5a). Under strongly acidic (pH 1.2) and mildly acidic (pH 4.5) conditions, a rapid release of CIP was observed, reaching approximately 80–90% within the first 3–5 h. In contrast, release at pH 6.8 was slower and more sustained, with ~30% released within 2 h and ~70% after 48–72 h. At pH 7.4, the release profile differed from that observed at pH 6.8, showing an initial rapid release (~60% within 1 h), followed by a gradual increase to ~80% between 8 and 12 h and approaching ~95% at 72 h.

These differences can be interpreted considering both drug properties and formulation characteristics. At pH 1.2 and 4.5, ciprofloxacin is predominantly protonated, which may increase its apparent solubility and favor partitioning into the aqueous phase, resulting in faster release. At pH 6.8, where the drug is predominantly in a zwitterionic or weakly ionized state, its reduced aqueous solubility may limit its transfer from the lipid matrix, contributing to a more sustained profile. However, this behavior is not solely governed by drug ionization. Differences between pH 6.8 and 7.4 suggest that medium composition and formulation structure also play a role, as both conditions fall within a similar ionization regime for ciprofloxacin. At pH 7.4, the relatively high release observed may be attributed to the contribution of drug fractions located near the nanoparticle surface or within less ordered lipid domains, which are more readily accessible to the aqueous phase. This interpretation is consistent with the initial burst observed in the first hour and suggests that drug distribution within the NLC matrix is an important factor governing release behavior. Therefore, the observed profiles likely result from a combination of pH-dependent drug solubility, medium composition, and structural organization of the lipid matrix, rather than a single controlling mechanism.

Several studies have reported CIP release profiles that are qualitatively comparable to those observed in the present work, even when using non-lipid matrices. For example, Sarwar et al. reported rapid CIP release (~80% within 2 h) from sodium alginate/poly(sodium 4-styrenesulfonate) films under acidic conditions, consistent with the fast release observed for NLC-CIP at low pH [40]. Similarly, Cassano et al. reported sustained release at pH 6.8, reaching ~50% cumulative release, in agreement with the partial and prolonged release observed under near-neutral conditions [41]. Despite differences in carrier composition, these trends highlight the dominant role of CIP physicochemical properties, particularly ionization state and solubility, in controlling release behavior.

In lipid-based systems, ciprofloxacin-loaded carriers consistently show pronounced pH-dependent, diffusion-controlled release. Nnamani et al. reported near-complete release (~98%) from NLCs under acidic or intestinal pH, depending on lipid composition [42]. In contrast, liposomal formulations containing nanocrystalline CIP exhibited limited release under buffered conditions (~25% at 24 h), with accelerated release in digestive-like environments due to lipid destabilization [43]. Youssef et al. observed gradual release at pH 7.4, with ~50–60% released within 4 h and ~82% over 24 h, attributed to drug entrapment within the solid lipid matrix [28]. Likewise, Almurshedi et al. showed that inhalable CIP-NLCs released only ~34% within 2 h and ~48% at 4 h, reaching ~80% after 10 h at pH 7.4 [44]. Overall, these findings situate the present NLC-CIP system within established lipid-based ciprofloxacin platforms and support a release behavior governed by drug physicochemical properties and diffusion constraints imposed by the lipid matrix.

Storage at 4 °C for 8 weeks did not alter the overall shape of the release profiles but resulted in a moderate reduction in the initial release rate across all pH conditions (Figure 5b). This effect is reflected in slightly flatter release curves for NLC-CIP(S), particularly during early time points. While the underlying mechanism was not directly investigated, this behavior is consistent with a potential redistribution of CIP within the lipid matrix or subtle structural rearrangements during storage, which may reduce the fraction of drug readily available for rapid release. Importantly, the extent of release at longer times remained comparable, indicating that storage did not compromise the overall release capacity of the system.

To obtain more mechanistic insight into the dominant release processes, kinetic analyses were performed by fitting the release data only up to the time at which each condition reached its respective plateau (Table 2). By focusing on the active release phase, this approach minimizes mathematical artifacts associated with late-stage saturation, facilitating a more rigorous interpretation of the governing transport mechanisms.

Kinetic analysis of the release profiles showed that the Weibull model provided the best overall fit across all pH conditions and formulations (R^2^ ≥ 0.96). Beyond its statistical performance, this model is particularly suitable for describing release from heterogeneous lipid systems, where multiple processes may contribute simultaneously to drug transport. The shape parameter (β < 1) observed in this study is consistent with a release profile characterized by a rapid initial phase followed by a progressive decrease in release rate, which aligns with the experimental observation of an initial burst followed by a slower diffusion phase. This behavior is commonly associated with drug release from partially disordered lipid matrices, such as those formed by Gelucire^®^ 44/14 and Miglyol^®^ 812, where domains with different degrees of organization may coexist and, consequently, different accessibility to ciprofloxacin. Although classical models such as Higuchi or Korsmeyer–Peppas also described portions of the release profiles, their applicability was limited to specific time intervals and did not adequately capture the full release behavior. This limitation was particularly pronounced under near-neutral conditions (pH 6.8–7.4), where lower *n* values were obtained for the Korsmeyer–Peppas model (e.g., ~0.29–0.38 at pH 6.8–7.4). These values suggest that the model may not be fully adequate to describe the transport dynamics in these environments. Nevertheless, this tendency has been previously reported, indicating a deviation from ideal diffusion behavior often caused by non-monodisperse particle populations and the structural complexity that can affect the apparent exponent [19]. Therefore, these values were interpreted cautiously and considered supportive rather than definitive evidence of a specific release mechanism. On the other hand, the *n* values obtained under acidic conditions fell within the anomalous transport range, which is consistent with release behavior involving diffusion coupled with additional matrix-related effects, although this interpretation should also be considered inferential.

In contrast, the Weibull model provided a consistent description across the entire dataset, supporting its use as a global descriptor of release kinetics in this system. However, it should be noted that model fitting alone does not demonstrate a specific release mechanism. Therefore, the interpretation of diffusion-dominated release in this work should be understood as consistent with the observed data and formulation characteristics, rather than as direct experimental proof. Notably, the apparent rate constants were consistently lower for NLC-CIP(S), supporting the view that storage reduces drug accessibility and effective matrix permeability during the initial stages of release.

From a formulation perspective, these results indicate that CIP release from NLCs is governed by the interplay between drug ionization, medium properties, and lipid matrix organization. The combination of rapid initial release and sustained diffusion over time suggests that the system is capable of modulating drug availability across different environments. At the same time, the dialysis method used in this study represents a simplified model that does not fully reproduce in vivo conditions, such as dynamic fluid composition or enzymatic effects. Likewise, stability was evaluated only under refrigerated conditions, which provided an initial and conservative assessment of formulation robustness but does not capture the full range of storage environments that may be relevant for broader pharmaceutical development. Additional stability studies under other storage conditions will be necessary to expand the pharmaceutical evaluation of the formulation. Therefore, the present findings should be interpreted as a comparative assessment of formulation performance under controlled conditions, providing mechanistic insight while acknowledging the limitations of the experimental model.

### 3.4. Antibacterial Activity Evaluation

**Figure 6:**
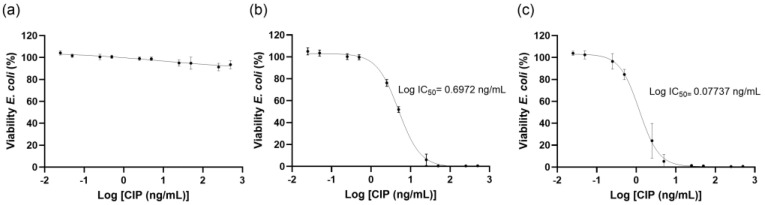
Dose–response curves of *Escherichia coli* viability following exposure to (**a**) NLC, (**b**) free CIP, and (**c**) NLC-CIP. Curves were fitted to determine the corresponding log IC_50_ values. Data represent mean ± standard deviation from three independent experiments, each performed in triplicate.

The antibacterial activity of NLC, NLC-CIP and free CIP was evaluated against *E. coli* (ATCC 25922) using dose–response analysis (Figure 6). Unloaded NLC showed no antibacterial activity at any tested concentration, with bacterial viability remaining above 100%, an IC_50_ > 0.008 mM, and a shallow Hill slope (−0.36), confirming the biological inertness of the carrier toward *E. coli* (Figure 6a). In contrast, CIP produced a clear dose-dependent inhibition, with an IC_50_ of 4.9 ng/mL and a steep Hill slope (−1.58) (Figure 6b). Notably, CIP encapsulation (NLC-CIP) preserved lower IC_50_, indicating improved antibacterial performance under the experimental conditions (Figure 6c), yielding a left-shifted dose–response curve and a lower IC_50_ of 1.2 ng/mL (Hill slope of −1.76). This corresponds to an approximately four-fold increase relative to CIP, however, this difference did not reach statistical significance (*p* = 0.01). This improvement can be attributed to formulation-mediated modulation of ciprofloxacin availability rather than intrinsic carrier activity and is consistent with sustained drug release and prolonged bacterial exposure, as reported for other lipid-based nanocarriers [44,45]. Overall, these results demonstrate that NLC can act as an effective platform for CIP delivery (1–10 ng/mL), preserving antibacterial activity while enabling controlled release and drug protection, thereby supporting their potential to improve the pharmaceutical performance of conventional antibiotics. Therefore, these results suggest improved antibacterial performance rather than a change in the intrinsic pharmacological activity of the drug.

### 3.5. Bacterial Growth Kinetics and Inhibitory Effect of Free and NLC-Encapsulated Ciprofloxacin

**Figure 7:**
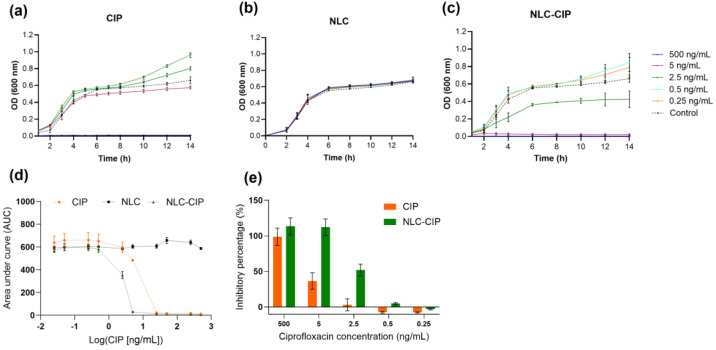
Bacterial growth kinetics of *Escherichia coli* (ATCC 25922) in the presence of CIP, NLC, and NLC-CIP. Panels (**a**–**c**) show representative growth curves for free CIP, NLC, and NLC-CIP, respectively. Panel (**d**) presents the area under the growth curve analysis, and panel (**e**) shows the corresponding percentage inhibition for CIP and NLC-CIP. Data are presented as mean ± SD from at least three independent experiments, each performed with three biological replicates. For unloaded NLC, particle concentration was adjusted to match the particle concentration present in each corresponding NLC-CIP formulation at each evaluated point.

Dynamic growth experiments revealed differences in the temporal antibacterial activity of the formulations (Figure 7a–c). The results showed that CIP inhibited bacterial growth at a concentration of 500 ng/mL, while at 5 ng/mL a slight reduction in final bacterial growth was observed. At lower concentrations, bacterial growth remained comparable to that of the control (Figure 7a). Unloaded NLCs showed no impact on bacterial growth, confirming carrier inertness (Figure 7b). In contrast, NLC-CIP induced marked growth inhibition at concentrations where CIP was ineffective, including complete growth inhibition under the experimental conditions at 5 ng/mL and partial inhibition at 2.5 ng/mL (Figure 7c). At the lowest concentrations tested, neither formulation affected bacterial growth under experimental conditions. In this way, encapsulating CIP into the NLC enables better modulation of CIP exposure and allows lower nominal concentrations to exert a measurable inhibitory effect on bacterial growth dynamics.

To quantitatively compare the overall inhibitory effects, the area under the growth curve (AUC) was calculated (Figure 7d), revealing distinct behaviors among formulations. While NLC-CIP and CIP showed comparable inhibitory profiles, NLC-CIP provided more consistent suppression across concentrations, indicating sustained ciprofloxacin activity. Consistently, growth inhibition analysis (Figure 7e) showed that at equivalent concentrations, NLC-CIP achieved >50% inhibition under conditions where CIP was markedly less effective.

The reduction in microbial growth may be explained by the combined effects of drug concentration, sustained release, and formulation-dependent drug presentation. In this context, NLC-CIP inhibited *E. coli* growth from the onset at a concentration of 5.0 ng/mL, whereas the same concentration of CIP produced a less pronounced effect. This difference may reflect a more favorable temporal profile of CIP availability in the nanoencapsulated system, rather than an increase in the intrinsic activity of the drug. In addition, the colloidal nature of the nanosystem may also contribute to this behavior, since nanoparticle-mediated drug presentation could facilitate local accumulation or retention of CIP near the bacteria–medium interface [46,47,48]. However, such interactions were not directly investigated in the present study and therefore should be considered a plausible, but still inferential, explanation.

Sustained release also appears to play a relevant role at lower concentrations. For example, at 2.5 ng/mL, NLC-CIP was able to suppress microbial growth after 6 h of exposure, whereas the same concentration of CIP produced growth behavior similar to the control. This observation is consistent with the prolonged release profile of the nanosystem and suggests that formulation-dependent modulation of drug exposure may contribute to the delayed but measurable antibacterial effect.

Taken together, these results suggest that the combination of sustained release and formulation-mediated control of CIP availability may allow lower nominal concentrations to reduce or inhibit microbial growth under the tested conditions. However, the precise mechanisms underlying these effects remain beyond the scope of the present study.

Bacterial growth rate analysis (Table 3) revealed significant reductions for NLC-CIP at concentrations ≥ 2.5 ng/mL, with stronger effects at the highest dose compared to CIP. In addition, doubling time analysis further supported improved antibacterial performance, with NLC-CIP increasing doubling time by nearly 200-fold at the highest concentration, compared to ~100-fold for CIP. Overall, these results indicate that NLC encapsulation enhances CIP efficacy by prolonging growth suppression and slowing bacterial proliferation rather than simply increasing static potency.

Reports on ciprofloxacin-loaded lipid systems are limited, and most studies rely on MIC or inhibition-zone data; nevertheless, these consistently show higher antibacterial potency for ciprofloxacin-loaded NLCs compared to free drug, in agreement with the present findings [29,30].

For other types of nanostructures, such as D-mannose–based microspheres, the findings similarly indicate that CIP exhibits enhanced efficacy when incorporated into a nanosystem. In this case, the authors report that the optimal performance occurred at 4.0 µg/mL; however, in the present work we report that the optimal concentration is an order of magnitude lower [41]. A comparable trend has also been reported for a range of inorganic nanocarriers, including chitosan-coated gold nanoparticles [49], iron oxide nanoparticles [50], and mesoporous silica-coated silver particles [51], all of which demonstrate improved antimicrobial activity when CIP is encapsulated into the nanosystem.

### 3.6. Cytotoxicity Assessment in HepG2 Cells

**Figure 8:**
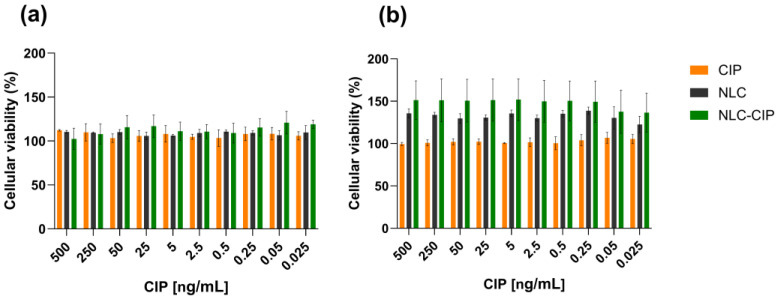
Cytotoxicity of NLC, NLC-CIP and CIP in HepG2 cells. (**a**) Cell viability after 24 h and (**b**) after 48 h of exposure to concentrations ranging from 0.025 to 500 ng/mL. Cellular metabolic activity was assessed by MTT assay and expressed as a percentage relative to untreated controls. No significant cytotoxic effects were detected for any treatment groups. Data are presented as mean ± standard deviation from three independent experiments, each performed in triplicate. For unloaded NLC, particle concentration was adjusted to match the particle concentration present in each corresponding NLC-CIP formulation at each evaluated point.

The cytocompatibility of CIP, NLC, and NLC-CIP was evaluated in HepG2 cells using the MTT assay after 24 h and 48 h of exposure. As shown in Figure 8a, no significant reductions in cell viability were detected after 24 h of treatment at any of the tested concentrations (0.025–500 ng/mL). Under all conditions, metabolic activity remained above 95%, indicating that neither the free drug nor the nanoparticle formulations compromised cellular viability at early time points. After 48 h of incubation (Figure 8b), cells exposed to free ciprofloxacin maintained viability levels close to or above 100% across the entire concentration range, confirming the absence of detectable cytotoxic effects. In contrast, cells exposed to unloaded NLCs or NLC-CIP exhibited an apparent increase in MTT activity relative to controls. This effect should be interpreted with caution. The MTT assay is based on the reduction of tetrazolium salts to formazan, and several studies have shown for lipid-based nanoparticles can interfere with this process through direct reduction of MTT, enhanced adsorption of formazan, physicochemical interactions with lipid structures or altered mitochondrial reduction, rather than true metabolic stimulation [52,53,54,55]. In particular, the lipophilic nature of formazan may promote its accumulation in lipid-rich environments, potentially leading to overestimation of metabolic activity. Therefore, the increased signal observed here may not necessarily reflect enhanced cell viability but could be partially attributed to assay-related artifacts.

Overall, these results demonstrate that both CIP and its nanoencapsulated formulation are well tolerated by HepG2 cells across the tested concentration range, supporting the biosafety of the NLC platform for further pharmaceutical and biological evaluation. Therefore, while the MTT assay provides useful information on cytocompatibility, its limitations in the context of nanoparticulate systems should be considered.

## 4. Conclusions

In this study, ciprofloxacin-loaded nanostructured lipid carriers were developed and systematically characterized as a nanoscale antibiotic delivery platform. Drug encapsulation induced predictable changes in physicochemical properties while maintaining a narrow size distribution, colloidal stability, and an amorphous drug state within the lipid matrix, supported by high encapsulation efficiency.

The release behavior of CIP was pH-dependent, with rapid release at pH 1.2, 4.5 and 7.4, while release was markedly slower at pH 6.8. Notably, this release pattern was maintained after refrigerated storage, indicating that storage does not compromise the underlying release mechanism. Kinetic analysis across all conditions was best described by the Weibull model, consistent with diffusion-dominated release but modulated by drug partitioning and matrix-related resistance.

Functionally, the NLC-based formulation improved antibacterial activity performance against *Escherichia coli*, as evidenced by lower IC_50_ values, reduced growth rates, and prolonged doubling times relative to free ciprofloxacin. The absence of intrinsic antibacterial activity for unloaded NLCs confirms that these effects arise from the drug–carrier interplay rather than from the nanosystem itself. Additionally, both unloaded and loaded NLCs demonstrated excellent cytocompatibility in HepG2 cells.

Taken together, our findings show that NLC-based encapsulation improves the antibacterial efficacy of CIP by reshaping drug exposure over time and modulating bacterial growth dynamics, while maintaining biocompatibility. The mechanistic insights gained highlight the relevance of release kinetics as a determinant of antibacterial efficacy and support the rational design of NLC platforms to improve antibiotic performance. Further studies in more complex infection models will be required to evaluate the translational potential of this approach.

## Figures and Tables

**Table 1:** Hydrodynamic diameter, PdI, and zeta potential of NLC and NLC-CIP measured by dynamic light scattering (DLS). Particle concentration was determined using nanoparticle tracking analysis (NTA). Values are reported as mean ± SD.

Sample	Hydrodynamic Diameter (nm)	PdI	Zeta Potential (mV)	Particle Concentration (Particle/mL)
NLC	116.0 ± 2.2	0.04 ± 0.01	−4.9 ± 0.2	1.5 × 10^17^
NLC-CIP	193.9 ± 1.7	0.04 ± 0.01	−1.2 ± 0.4	9.9 × 10^16^

**Table 2:** Kinetic parameters obtained by fitting the Weibull, power law (Korsmeyer–Peppas), Higuchi, zero-order, and first-order models to the ciprofloxacin release profiles from NLC-CIP(F) and NLC-CIP(S) formulations. The analysis was restricted to the active release phase and performed up to the time at which each condition reached its respective plateau.

Formulations	Mathematical Models									
	Weibull		Power Law		Higuchi		Zero-Order		First-Order	
	β	R^2^	*n*	R^2^	k_H_ (% h^−0.5^)	R^2^	k_0_ (% h^−1^)	R^2^	k_1_ (h^−1^)	R^2^
NLC-CIP(F) at pH 1.2	0.85	0.99	0.62	0.96	50.3	0.95	25.9	0.42	0.92	0.97
NLC-CIP(F) at pH 4.5	0.76	0.98	0.61	0.89	45.4	0.96	23.6	0.49	0.80	0.95
NLC-CIP(F) at pH 6.8	0.45	0.98	0.32	0.97	14.6	0.67	NA	NA	0.08	0.62
NLC-CIP(F) At pH 7.4	0.41	0.96	0.38	0.82	28.6	0.44	NA	NA	NA	NA
NLC-CIP(S) at pH 1.2	0.67	0.99	0.59	0.95	26.1	0.80	NA	NA	0.22	0.88
NLC-CIP(S) at pH 4.5	0.62	0.98	0.53	0.94	25.0	0.81	NA	NA	0.19	0.85
NLC-CIP(S) at pH 6.8	0.44	0.98	0.29	0.97	14.4	0.70	NA	NA	0.07	0.61
NLC-CIP(S) At pH 7.4	0.5	0.97	0.34	0.98	27.4	0.94	9.6	0.29	0.26	0.78

NA: indicates that the model was not applicable due to poor fitting quality and lack of physical meaning of the estimated parameters.

**Table 3:** Growth kinetics parameters of *Escherichia coli* following exposure to CIP, NLC, and NLC-CIP. The table summarizes the bacterial growth rate (k, h^−1^) and doubling time (Dt, h) across a ciprofloxacin concentration range from 0.5 µg/mL to 0.025 ng/mL). “C” represents control.

		CIP (ng/mL)					NLC-CIP (ng/mL)				
Parameters	C	500	5	2.5	0.5	0.25	500	5	2.5	0.5	0.25
k (h^−1^)	0.23	0.002	0.21	0.25	0.27	0.27	0.001	0.006	0.16	0.28	0.28
Dt (h)	3.0	346.0	3.3	2.8	2.6	2.6	693.0	115.0	4.3	2.5	2.5

## Data Availability

The original contributions presented in this study are included in the article. Further inquiries can be directed to the corresponding authors.
